# DNA Adsorption Studies of Poly(4,4′-Cychlohexylidene Bisphenol Oxalate)/Silica Nanocomposites

**DOI:** 10.3390/ma12071178

**Published:** 2019-04-11

**Authors:** Aisha Nawaf Al balawi, Nor Azah Yusof, Sazlinda Kamaruzaman, Faruq Mohammad, Helmi Wasoh, Hamad A. Al-Lohedan

**Affiliations:** 1 Department of Chemistry, Faculty of Science, Universiti Putra Malaysia, Serdang 43400 UPM, Malaysia; an.albalawi@ut.edu.sa (A.N.A.b.); sazlinda@upm.edu.my (S.K.); 2 Haql College, University of Tabuk, Tabuk 71491, Saudi Arabia; 3 Institute of Advanced Technology, Universiti Putra Malaysia, Serdang 43400 UPM, Malaysia; 4 Surfactants Research Chair, Chemistry, College of Science, King Saud University, Riyadh 11451, Saudi Arabia; hlohedan@ksu.edu.sa; 5 Faculty of Biotechnology and Biomolecular Science, Universiti Putra Malaysia, Serdang 43400 UPM, Malaysia; helmi_wmi@upm.edu.my

**Keywords:** poly(4,4′-cyclohexylidene bisphenol oxalate), surface modification, silicon, nanocomposite, DNA extraction, polymerization

## Abstract

The present study deals with the synthesis, characterization, and DNA extraction of poly(4,4′-cyclohexylidene bisphenol oxalate)/silica (Si) nanocomposites (NCs). The effects of varying the monomer/Si (3.7%, 7%, and 13%) ratio towards the size and morphology of the resulting NC and its DNA extraction capabilities have also been studied. For the NC synthesis, two different methods were followed, including the direct mixing of poly(4,4′-cyclohexylidene bisphenol oxalate) with fumed Si, and in situ polymerization of the 4,4′-cyclohexylidene bisphenol monomer in the presence of fumed silica (11 nm). The formed NCs were thoroughly investigated by using different techniques such as scanning electron microscopy (SEM), fourier transform infrared (FTIR) spectroscopy, differential scanning calorimetry (DSC), thermogravimetric analysis (TGA), powdered X-ray diffraction (XRD), and Brunauer–Emmett–Teller (BET) analysis where the results supported that there was the successful formation of poly(4,4′-cyclohexylidene bisphenol oxalate)/Si NC. Within the three different NC samples, the one with 13% Si was found to maintain a very high surface area of 12.237 m^2^/g, as compared to the other two samples consisting of 7% Si (3.362 m^2^/g) and 3.7% Si (1.788 m^2^/g). Further, the solid phase DNA extraction studies indicated that the efficiency is strongly influenced by the amount of polymer (0.2 g > 0.1 g > 0.02 g) and the type of binding buffer. Among the three binding buffers tested, the guanidine hydrochloride/EtOH buffer produced the most satisfactory results in terms of yield (1,348,000 ng) and extraction efficiency (3370 ng/mL) as compared to the other two buffers of NaCl (2 M) and phosphate buffered silane. Based on our results, it can be indicated that the developed poly(4,4′-cyclohexylidene bisphenol oxalate)/Si NC can serve as one of the suitable candidates for the extraction of DNA in high amounts as compared to other traditional solid phase approaches.

## 1. Introduction

In recent years, polymer nanocomposites (NCs) have attracted attention from the researchers both fundamental and applications point of view because of their attractive properties (mechanical, thermal, optical, electrical, structural, and biomedical), where they can serve as potential candidates in many different areas of industrial product development [1,2]. In comparison to conventional materials, polymer NCs often display superior properties such as stiffness, strength, solvent dispersibility, oxidative stability, thermal resistance, electrical conductivity, and biodegradability [3]. The physical and surface properties of NCs are greatly influenced by the interfacial adhesion between the organic compound and nanoparticles (NPs) dispersing in the polymer matrix. Layered silicate or silica (Si) foam NPs are also among the leading nanoscale materials in research and development, although polymer NCs have nano-reinforcements such as nanoclays, graphite platelets, and carbon nanotubes. These materials are widely used because of their high surface energy, mild reactivity, and well controllable chemical properties. 

Si is available from nature mainly in the form of sand or quartz crystals; however, it is generally manufactured as crystals, fused quartz, colloidal silica, fumed silica, silica gel, and aerogel. The various forms of low-dimensional Si include NCs with ring, chain, cage, and tubular structures [4,5,6]. These Si nanostructures contain the rings of Si–O (two-, three-, four- and six-membered) accompanied by the presence of non-bridging oxygen atoms [7,8]. The tubular and the layered configurations have attained particular attention both theoretically and experimentally through these nanostructures. The layered silicates consist of thin layers that are always bound together with counter-ions and the basic blocks are tetrahedral sheets, in which four oxygen atoms surround Si and octahedral sheets with eight oxygen atoms surround the metal (such as aluminum) [9]. 

The layered silica has a high aspect ratio (10–1000) and layer thickness in the order 1 nm. Therefore, a small percentage increase in weight of layered silica results in a much higher surface area when they are dispersed throughout the polymer matrix. It has been shown that fumed silica aggregates have two different fractal dimensions [10]. The mesostructured (hexagonal) fumed Si is a unit of matter characterized by softness and flexibility, a high ratio of length to the thickness, and chemical and thermal stability [11]. These properties of fumed silica make them quite competitive for specific applications. These particles are bonded with polymers, resulting in high viscosity properties of polymers [12]. Three different types of polymer/Si NCs are intercalated NCs, flocculated NCs, and exfoliated NCs [13]. 

Si NPs are associated with dispersion in many thermosetting, thermoplastic, elastomers, natural, and biodegradable polymers. This procedure is carried on the basis of a variety of approaches including direct mixing of polymer and NPs [14], intercalation of polymer or prepolymer from solution [15] including melt intercalation [16], sol-gel method (in situ template synthesis (sol-gel technology)) [17], and in situ polymerization method [18,19]. The properties of NCs depend on the properties of individual components and other parameters. These parameters include certain processes used in NC fabrication, the degree of mixing of the two phases, the volume fraction of NPs, the types of filler materials and their orientations, type of adhesion at the matrix interface, morphology of the system, NPs characteristics, size and shape of NPs, and nature of the interphase developed at the matrix interface materials [20].

The currently available methods used for the DNA extraction suffers from the issues like high costs associated with the equipment, repetition and reproducibility of same results, prolonged time periods, complex protocols, obligation of qualified personnel etc. [21,22,23,24,25,26,27,28]. To address some of these issues, the adsorption procedures, which have the advantage of nanotechnology, and separation science principles were applied. This includes the use of many different adsorbent materials like glass particles [21], silica-based matrices [22,23], magnetic NPs [24], diatomaceous earth [25], anion-exchange materials [26], and cellulose matrices [27,28]; however, each material has its own advantages and disadvantages that are described briefly in Table 1. From the perspective that the effectiveness, equipment portability, and analysis costs have to be equally poised, the polymeric NCs are considered to serve as the suitable materials for the extraction of DNA and that too within many different kinds of polymer materials, the hybrid scaffolds made up of poly(bisphenol Z oxalate) are emerging as finest adsorbents for the selective and high efficiency DNA extraction [29].

By keeping in view of the selective and specific properties offered by poly(bisphenol Z oxalate) for the high efficiency DNA extraction and Si NP’s solid supported properties, the present study aims to develop an ideal NC system that has a very high DNA extraction efficiency. Our earlier study deal with the synthesis and characterization of pure poly(4,4′-cyclohexidene bisphenol oxalate) for the DNA extraction [29]. Further, to improve the DNA adsorption capabilities of the same poly(4,4′-cyclohexidene bisphenol oxalate) by means of influencing the inbuilt properties, the present study was carried where the polymer was used to form the composite with that of Si NPs. In addition, this study investigates the effects of nanosilica on the polymeric matrix when added at differential ratios of fumed Si to the matrix. Formed NCs were characterized thoroughly in terms of their surface area, structure, shape, porosity, surface morphology, etc. Finally, the NCs were applied to extract DNA from a solution mixture, intending to determine the key factors related to the ratio of Si NCs and other processing conditions for high efficiency DNA extraction.

## 2. Material and Methods

### 2.1. Materials

The DNA solution used was ssDNA (single-stranded deoxyribonucleic acid), purchased from Sigma-Aldrich company (D7290, Selangor D.E, Malaysia). The exposure of ssDNA to sonication shears the large molecular weight DNA to produce fragments in a size range from 587 to 831 base pairs with a concentrated solution of 9–12 mg/mL DNA. Fumed silica NPs, 4-dimethyl aminopyridine (DMAP) with the particle size of 11 nm were purchased from Sigma-Aldrich company. All other solvents, including tetrahydrofuran (THF), chloroform, ethanol, and other buffers were also purchased from Sigma-Aldrich company. All the reagents used were of the highest grade and were received and utilized with no further purification. Poly(4,4′-cyclohexylidene bisphenol oxalate) is a white powder that was prepared by the condensation polymerization method [30].

### 2.2. Preparation of Poly(4,4′-Cyclohexylidene Bisphenol Oxalate)/Si NC

The methods used for synthesis of poly(4,4′-cyclohexylidene bisphenol oxalate)/Si NCs were direct mixing and in situ polymerization.

#### 2.2.1. Direct Mixing of Polymer and Si NPs (Solution-Mixing Method)

A predetermined amount of Si NPs were dispersed in 141.5 g of chloroform and subjected to ultra-sonication for 30 min in a water bath. After this period, poly(4,4′-cyclohexylidene bisphenol oxalate) was dissolved in the chloroform/Si NPs solution at room temperature for 24 h with vigorous stirring. The final concentration of poly(4,4′-cyclohexylidene bisphenol oxalate) in chloroform was 2.1 wt.% and the Si NPs content was 40 wt.% based on the polymer matrices. The solution was then co-precipitated in 500 mL of methanol, washed with deionized water, and filtered. The sample was dried at 70 °C in a hot air oven for 24 h to obtain the powdered form of polymer/Si NCs [1,31].

#### 2.2.2. In Situ Polymerization Method

The poly(4,4′-cyclohexylidene bisphenol oxalate)/Si NCs were synthesized by an in situ condensation polymerization of monomer bisphenol Z with diol of oxalyl chloride. At first, a solution of oxalyl chloride (0.02 mol, 2.525 g) and dried THF (20 mL) were added drop-wise to a mixture of monomer 4,4-cyclohexylidine bisphenol (0.02 mol, 5.367 g). Certain amounts of Si NPs and pyridine (0.12 mol) in dried THF (40 mL) with catalyst DMAP were also added in the solution and maintained at 0–5 °C using an ice bath. The addition was followed by the stirring of reaction mixture for 1 h at 0–5 °C temperature. The ice bath was removed and the mixture was allowed to stand for another 24 h. After that, the reaction mixture was diluted with chloroform (100 mL) and washed with water. Finally, methanol was added drop-wise and the solvent was evaporated slowly to the organic portion that resulted in the precipitation of product. The precipitation was collected by filtration and dried at 70 °C under vacuum to obtain the white powder. The measured values are presented in Table 2.

Polymer yield = 100 × amount of polymer NC produced (g)/amount of bisphenol Z and oxalyl chroide (g) charged

### 2.3. Studies of DNA Extraction

**Figure 1:**
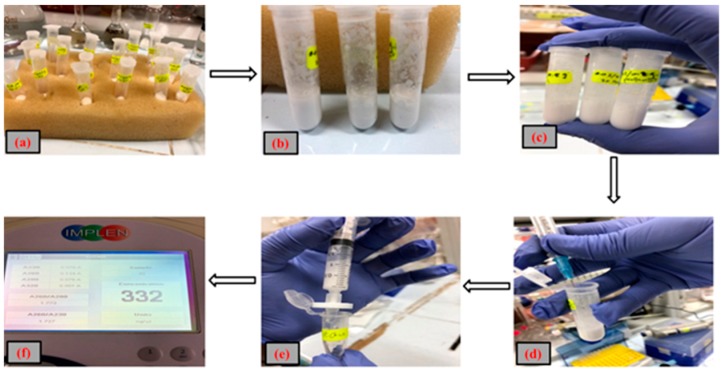
Schematic representation of the process to extract DNA using our polymer/Si NC. As shown in the figure, the following steps were carried out: (**a**) poly(4,4′-cyclohexylidene bisphenol oxalate)/Si(13 wt.%) NCs, weighing in the order of 0.2 g, 0.01g, and 0.02 g, were inserted into Eppendorf tube; (**b**) we added binding buffer and DNA solution to a total volume of 500 µL; (**c**) incubation for 10 min; (**d**) we took out the solution; (**e**) separation of elution from the polymer NC; and (**f**) the efficiency and purity of the extracted DNA was assessed from the eluted buffer by NanoPhotometer™.

For DNA analysis, 2 M of guanidine hydrochloride (GuHCl) in 96% ethanol (EtOH), 2 M NaCl solution, and phosphate buffered silane (PBS, 5 M GuHCl in 30% propanol) were used as binding buffers to measure the binding capacity of poly(4,4′-cyclohexylidene bisphenol oxalate)/Si (13.0 wt.%) NC. A 200 µL of DNA solution (20 µL DNA and 180 µL of deionized water) was mixed with 300 µL binding buffer. The poly(4,4′-cyclohexylidene bisphenol oxalate)/Si (13 wt.%) NCs, with weights in the order of 0.2 g, 0.01 g, and 0.02 g, were inserted into Eppendorf tube including binding buffer and DNA solution with a total volume of 500 µL, followed by incubation for 10 min. The solution was taken out and washed with 70% EtOH to clean all the salt from the surface using a pipette. In the next step, 1000 µL of elution buffer was added to the tubes and it was incubated for another 5 min, followed by the separation of elution from the polymer. The efficiency and purity of the extracted DNA was assessed from the eluted buffer. Figure 1 shows the process of DNA extraction by making use of the synthesized poly(4,4′-cyclohexylidene bisphenol oxalate)/Si NCs.

### 2.4. Purity and Yield Analysis of Extracted DNA

The absorbance ratio between 260 nm and 280 nm was measured using nanophotometer device that range from 1.8 to 2.0 for high purity DNA. Equations (1) and (2) were used to calculate the total yield of DNA purification and the extraction, respectively.

(1) $$\begin{array}{l} Total\;yield\;of\;DNA\;purification=\;A\;final\;elution\;of\;the\;solution’s\;volume\;\times \;\\ DNA\;concentration\;\left(ng/\mu L\right) \end{array}$$

(2) $$\begin{array}{l} Extraction\;efficiency=\;Total\;DNA\;yield/total\;DNA\;amount\;\\ \left(ng\;input\;DNA\;volume,\;\mu L\right) \end{array}$$

### 2.5. Characterization of the Poly(4,4′-Cyclohexylidene Bisphenol Oxalate)/Si NC

The changes in functional groups of the polymer at different stages of its formation were characterized by Fourier transform infrared (FTIR) spectroscopy, where PerkinElmer Spectrum 100 FTIR instrument (Shelton, CT, USA) was applied. For the analysis, the samples were recorded in the range 4000–450 cm^−1^ using KBr pellets. The thermogravimetric analysis (TGA), the differential thermogravimetry (DTG), and differential scanning calorimetry (DSC) analysis were used to analyze the thermal stability of the polymer NC at a temperature range of 60–700 °C. Mettler Toledo instrument (Columbus, OH, USA) was applied for the testing. The changes in the surface morphology and other physical properties of the polymer composite were characterized by scanning electron microscopy (SEM), for which a Module NOVA NANOSEM 230-FE 1TM instrument (FEI company, Hillsboro, OR, USA) was employed. Powdered X-ray diffraction (XRD) analysis was performed on XRD 6000 instrument (Shimadzu, Kyoto, Japan) where the interlayers between polymer and Si NPs were studied for the elemental analysis. The XRD patterns were measured based on the conditions that include Cu target, 30 kV voltage, 30 mA current, scan range of 2–60, scan speed 2 deg/min, 0.02 deg sampling pitch, and 0.6 sec preset time. The d-spacing was calculated for the large peak(s) in the respective curves from Bragg’s equation, *nλ = 2d Sinθ*. The surface area of poly(4,4′-cyclohexylidene bisphenol oxalate)/Si NC was analyzed by means of BET (Brunnet–Emmet–Teller) and BJH (Barrett–Joyner–Halenda) methods, for which the Autosorb 1 Module Quantachrome instrument (Boynton Beach, FL, USA) was applied. The UV-Vis spectroscopic analysis was studied by the Nanodrop 2000 spectrophotometer (Thermo Fisher Scientific, Waltham, MA, USA). A nanophotometer device module was used to study the DNA adsorption.

## 3. Results and Discussion

**Figure 2:**
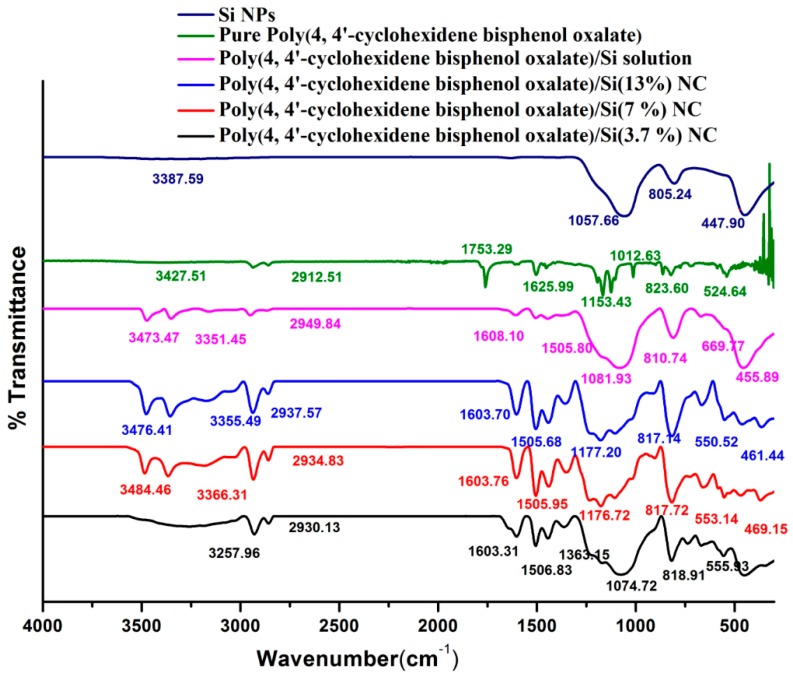
FTIR spectral comparison different ratios of Si in poly(4,4′-cyclohexylidene bisphenol oxalate/Si NC, with that of pure forms of poly(4,4′-cyclohexylidene bisphenol oxalate) and Si NPs.

The modification of polymer with Si NPs was analyzed by FTIR spectroscopy, as shown in Figure 2. The bands at 3366.31 cm^−1^ and 2949.84 cm^−1^ were attributed to the CH_2_ asymmetric and symmetric vibrations of the poly(4,4′-cyclohexylidene bisphenol oxalate/Si NC [32]. The results show that 3257.96 cm^−1^ was assigned to the C–H stretching in the cyclohexane of polymer/Si(3.7%), 3182.34 cm^−1^ of the C–H stretching of polymer/Si(7%), 3173.58 cm^−1^ for the C–H stretching of polymer/Si(13%), and 3157.59 cm^−1^ to C–H stretching of the polymer/Si solution. The spectra of the NCs displayed both the characteristic absorption bands at 1057–805.24 cm^−1^ of S–O stretching vibration of SiO_2_ (Table 3). The C=C group of pure polymer and polymer/Si NCs was observed to be around 1626–1603 cm^−1^.

**Figure 3:**
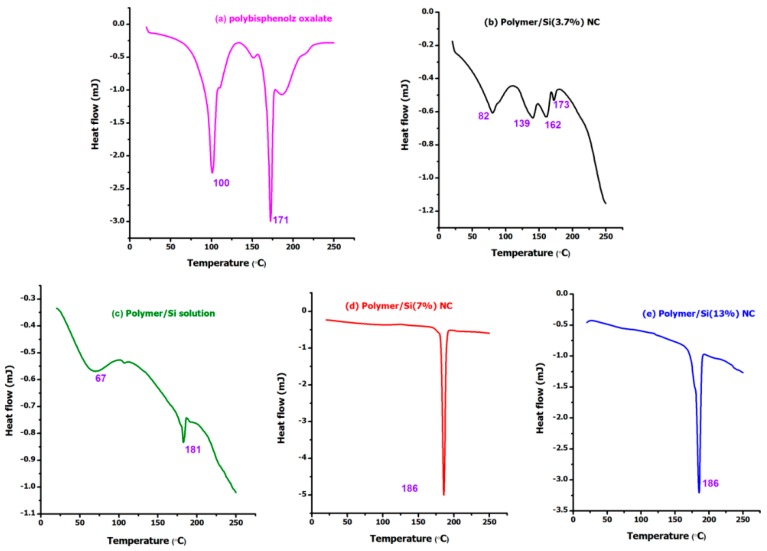
Comparison of the DSC thermograms of poly(4,4′-cyclohexylidene bisphenol oxalate) and poly(4,4′-cyclohexylidene bisphenol oxalate/Si NCs: (**a**) Pure poly(4,4′-cyclohexylidene bisphenol oxalate); (**b**) poly(4,4′-cyclohexylidene bisphenol oxalate)/Si(3.7%) NC; (**c**) poly(4,4′-cyclohexylidene bisphenol oxalate)/Si solution,); (**d**) poly(4,4′-cyclohexylidene bisphenol oxalate)/Si(7%) NC; and (**e**) poly(4,4′-cyclohexylidene bisphenol oxalate)/Si(13%) NC.

Figure 3 shows the DSC thermograms of poly(4,4′-cyclohexylidene bisphenol oxalate)/Si NC containing 0.005 mol, 0.01 mol, and 0.02 mol of SiO_2_. From the analysis, it can be observed that three of the samples showed a peak for loss of water molecules (around 100 °C) with an exception to the two samples, namely, poly(4,4′-cyclohexylidene bisphenol oxalate)/Si(7%) and poly(4,4′-cyclohexylidene bisphenol oxalate)/Si(13%) NCs, meaning that there are no water molecules or moisture to these samples. For the poly(4,4′-cyclohexylidene bisphenol oxalate) sample, the observation of two peaks (100 °C and 171 °C) could be due to the result of melt/recrystallization process or phase-separated structure formation. The two endothermic peaks observed are usually due to the formation of imperfect/unstable crystals, whereas the higher temperature peaks are regarded as perfect and stable ones [33,34]. The various degradation peaks for the samples observed in the DSC analysis are compared in Figure 3. In Figure 3b, the poly(4,4′-cyclohexylidene bisphenol oxalate)/Si(3.7%) sample containing 0.005 mol of silica NPs appeared with four different degradation peaks, that is, 82 °C, 139 °C, 162 °C, and 173 °C, which can be related to melting point (Tm) temperatures of the semi-crystalline copolymer and amorphous NC. These peaks are associated with pseudo-melting temperature due to the mixture of chain polymer with Si NPs without interaction which are able to form a liquid crystalline structure. From the comparison of results, it is apparent that the increased ratio of Si content in the polymer matrix increased the degradation temperature of the lower peak and this may be due to the occurrence of the melt/recrystallization process. As a result, the poly(4,4′-cyclohexylidene bisphenol oxalate)/Si NC increased its melting point to 173 °C (Figure 3b), 181 °C (Figure 3c), 186 °C (Figure 3d) and 186 °C (Figure 3e) with increasing Si content. However, the melting/degradation temperatures of poly(4,4′-cyclohexylidene bisphenol oxalate)/Si(7%) and poly(4,4′-cyclohexylidene bisphenol oxalate)/Si(13%) showed almost no change. The total signal indicates an endothermic peak above 67 °C in poly(4,4′-cyclohexylidene bisphenol oxalate)/Si solution sample. The observation of increased degradation temperature for the polymeric matrix with increase of Si loading can be due to the extra stability offered by the nanosilica to withstand from the heat induced stress, thereby supporting the composite formation. 

**Figure 4:**
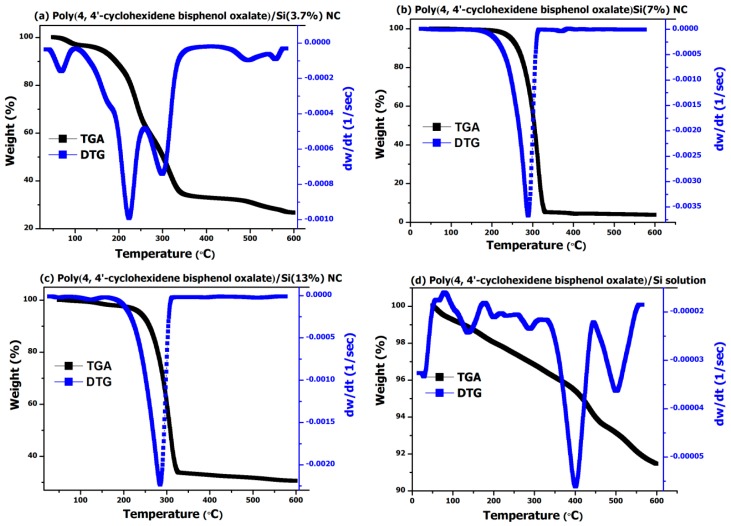
TGA and DTG analysis for poly(4,4′-cyclohexylidene bisphenol oxalate)/Si NCs: (**a**) poly(4,4′-cyclohexylidene bisphenol oxalate)/Si(3.7%), (**b**) poly(4,4′-cyclohexylidene bisphenol oxalate)/Si(7%), (**c**) poly(4,4′-cyclohexylidene bisphenol oxalate)/Si(13%) NC, and (**d**) poly(4,4′-cyclohexylidene bisphenol oxalate)/Si solution mixture.

The TGA and DTG analysis performed on poly(4,4′-cyclohexylidene bisphenol oxalate)/Si NCs showed that the polymer filled with Si NPs exhibited improved thermal stability (Figure 4). For the TGA analysis, for example, the onset of thermal degradation process for poly(4,4′-cyclohexylidene bisphenol oxalate)/Si(3.7%) NC sample (Figure 4a) exhibited three important weight loss regions, that is, one up to 120 °C with a total weight loss of around 5%, the second in the region of 120–350 °C with sample weight loss of 66%, and the third around 350–600 °C range with a loss of 78%. However, with an increase in Si content to 7% (Figure 4b) and 13% (Figure 4c), we observed only one major weight loss region around 220–335 °C and a total weight loss of more than 65% due to the degradation or recrystallization of the polymer. In a similar way, for the poly(4,4′-cyclohexylidene bisphenol oxalate)/Si solution (Figure 4d), the thermal stability was further increased and we observed only a 9% weight loss over a region of up to 600 °C, thereby confirming the ability of Si NPs towards enhancing thermal stability.

One integral in the DTG analysis was observed for poly(4,4′-cyclohexylidene bisphenol oxalate)/Si solution (Figure 4d) having the sample loss of 0.053 mg around the degradation temperature of 432 °C. Similarly, for the two composite samples (Figure 4b,c), the poly(4,4′-cyclohexylidene bisphenol oxalate)/Si(7%) NC and poly(4,4′-cyclohexylidene bisphenol oxalate)/Si(13%) exhibited sample losses of 3.68 mg and 3.09, which correspond with the degradation process occurring around 313 °C and 307 °C, respectively. From further comparison of results presented in Figure 3b–d, it can be seen that the polymer degradation or recrystallization process is occurring before 350 °C as observed by the tree different peaks having the sample loss of 0.16 mg (74 °C), 0.69 mg (169 °C), and 5.96 mg (334 °C). Thus, from the analysis of results obtained from thermal degradation studies, it is proved that the synthesized NCs have relatively high thermal stability and Tm (degradation temperature and weight loss). Such a significant improvement in the degradation temperature of the synthesized NCs is due to the homogeneous dispersion of the silicate NPs that got localized into the sites of the functional copolymer matrix. 

**Figure 5:**
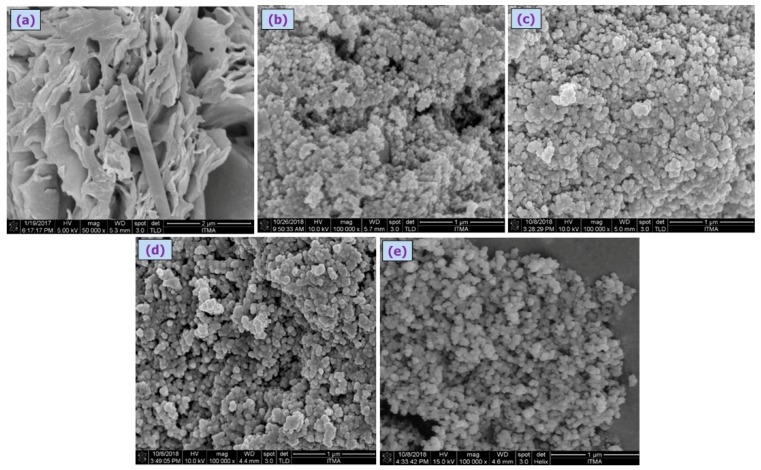
SEM images of (**a**) poly(4,4′-cyclohexylidene bisphenol oxalate), (**b**) poly(4,4′-cyclohexylidene bisphenol oxalate)/Si(3.7%) NC, (**c**) poly(4,4′-cyclohexylidene bisphenol oxalate)/Si(7%) NC, (**d**) poly(4,4′-cyclohexylidene bisphenol oxalate)/Si(13%) NC, and (**e**) poly(4,4′-cyclohexylidene bisphenol oxalate)/Si solution mixture containing 40 wt.% NC.

The poly(4,4′-cyclohexylidene bisphenol oxalate)/Si NCs characterized by the SEM analysis are shown in Figure 5a–c, where the particles were observed to be of narrow size distributed in smooth spherical shape (Figure 5b–e). The average diameters of poly(4,4′-cyclohexylidene bisphenol oxalate)/Si(3.7%), poly(4,4′-cyclohexylidene bisphenol oxalate)/Si(7%), poly(4,4′-cyclohexylidene bisphenol oxalate)/Si(13%) NC, and poly(4,4′-cyclohexylidene bisphenol oxalate)/Si solution were observed to be 67.8 nm, 61.5 nm, 60.6 nm, and 55.9 nm, respectively. The average particle size of polymer before modification in (Figure 5a) was 162.45 nm with a large gap and rough surface. 

**Figure 6:**
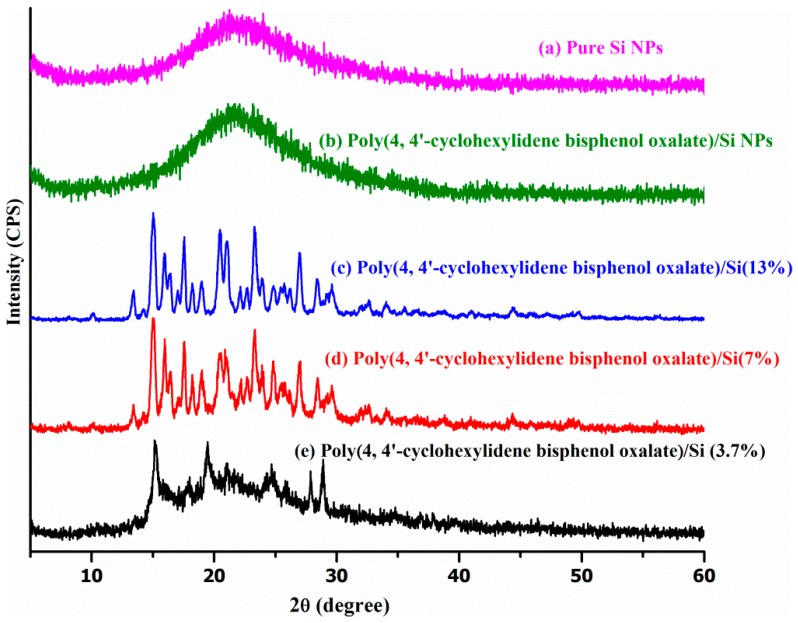
Comparison of the powdered XRD patterns of poly(4,4′-cyclohexylidene bisphenol oxalate), poly(4,4′-cyclohexylidene bisphenol oxalate)/Si(3.7%), poly(4,4′-cyclohexylidene bisphenol oxalate)/Si(7%), poly(4,4′-cyclohexylidene bisphenol oxalate)/Si(13%), and poly(4,4′-cyclohexylidene bisphenol oxalate)/Si sol mixing, along with fumed pure Si.

Powdered XRD technique is the most indicative technique investigating interactions between the Si layers and the polymer. The XRD patterns of pure polymer powder and the poly(4,4′-cyclohexylidene bisphenol oxalate)/Si NCs prepared in this study are shown in Figure 6. The peak at 2θ of 29° can be attributed to the basal spacing (interlayer gap) (~3.069 Å) from the polymer used for the composite formation, while the poly(4,4′-cyclohexylidene bisphenol oxalate)/Si NC at 2θ peak was shifted to a lower value around 15°. The interlayer gap of polymer was increased from 3.069 to 5.850 Å, 5.843 Å and 5.802 Å for poly(4,4′-cyclohexylidene bisphenol oxalate)/Si(13%), poly(4,4′-cyclohexylidene bisphenol oxalate)/Si(7%), and poly(4,4′-cyclohexylidene bisphenol oxalate)/Si(3.7%), respectively. Table 4 shows the XRD results that showed an increase in the basal spacing of poly(4,4′-cyclohexylidene bisphenol oxalate)/Si solution mixing at 4.033 Å. However, this value was lower than the basal spacing of poly(4,4′-cyclohexylidene bisphenol oxalate)/Si NC, which was prepared by the in situ polymerization technique. This reveals the formation of intercalated structures due to the interaction between polymer chains of Si NPs. This expansion of the polymer basal spacing was due to the favorable interactions between the Si NPs and the polymer groups. The best interpretations about the relationship between intercalated regions and the amount of Si NPs have been reported in previous studies, and they confirm that there is a successful bonding between the polymer groups and Si particles [35,36]. 

**Figure 7:**
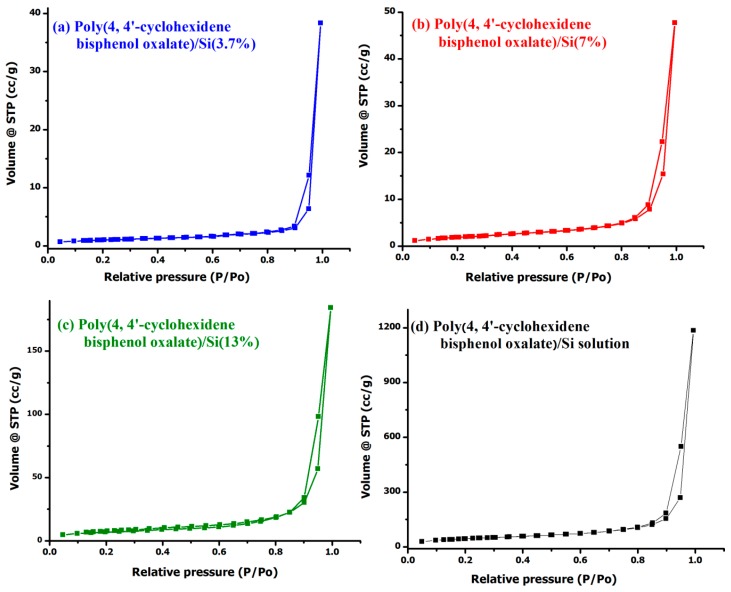
Brunnet–Emmet–Teller (BET) results for poly(4,4′-cyclohexylidene bisphenol oxalate)/Si NCs having wt.% of 3.7%, 7%, 13% and poly(4,4′-cyclohexylidene bisphenol oxalate)/Si solution.

The surface area of poly(4,4′-cyclohexylidene bisphenol oxalate)/Si NCs was measured by BET analysis, where the N_2_ gas was applied as adsorptive and the bath was maintained at 77.251 K temperature. Figure 7 shows the N_2_-adsorption-desorption isotherms curves for poly(4,4′-cyclohexylidene bisphenol oxalate)/Si(3.7%) NC, poly(4,4′-cyclohexylidene bisphenol oxalate)/Si(7%) NC, poly(4,4′-cyclohexylidene bisphenol oxalate)/Si(13%) NC, and poly(4,4′-cyclohexylidene bisphenol oxalate)/Si solution [37]. As classified by the IUPAC (International union of pure and applied chemistry), this type of adsorption isotherm demonstrates the presence of a weak adsorptive adsorbent interaction between the N_2_ molecules and the polymer membrane. Table 5 reports three values of the surface area including the pore volume-pore radius for all the investigated samples. The values in the table were calculated by the BJH and BET method. It was observed that the N_2_-mediated BET surface area of the poly(4,4′-cyclohexylidene bisphenol oxalate)/Si NCs increased with an increased ratio of Si in the polymer between 1.79–76.95 m^2^/g. The values of BJH adsorption and desorption surface were observed at higher values of 120.35 m^2^/g and 151.62 m^2^/g for poly(4,4′-cyclohexylidene bisphenol oxalate)/Si solution. This enhancement in the surface area improves the properties of the poly(4,4′-cyclohexylidene bisphenol oxalate)/Si NCs. 

### Extraction Efficiency and Purity of ssDNA

**Figure 8:**
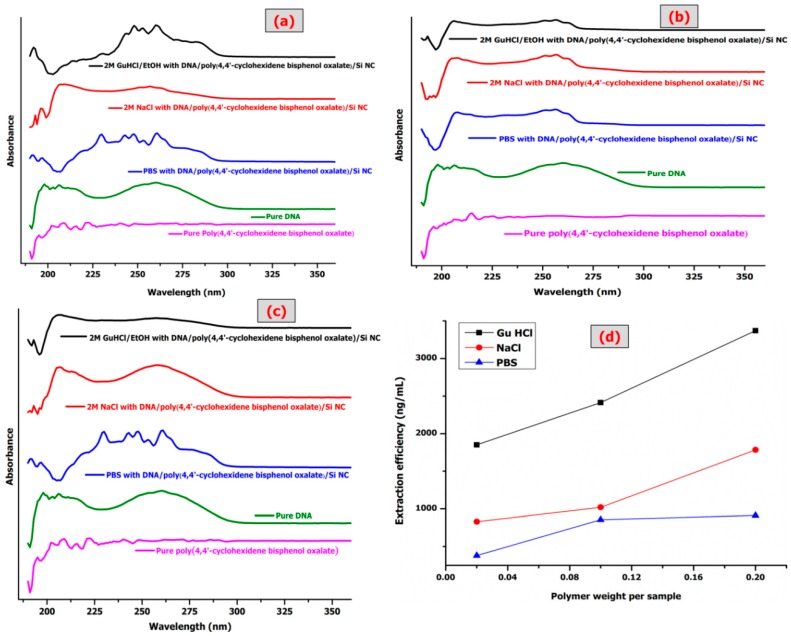
UV-Vis spectral comparison of DNA bonded with (**a**) 0.2 g of poly(4,4′-cyclohexylidene bisphenol oxalate)/Si(13%) NC, pure poly(4,4′-cyclohexylidene bisphenol oxalate), and pure DNA; (**b**) 0.1 g of poly(4,4′-cyclohexylidene bisphenol oxalate)/Si(13%) NC; (**c**) 0.02 g of poly(4,4′-cyclohexylidene bisphenol oxalate)/Si(13%) NC; and (**d**) shows a comparison of DNA extraction efficiency against changes in the weights of polymer (0.02 g, 0.1 g, and 0.2 g) and binding buffer solutions (GuHCl/96% EtOH, PBS, and NaCl).

Figure 8 shows three different binding buffers with three different weights of poly(4,4′-cyclohexylidene bisphenol oxalate)/Si NCs to measure the ssDNA extraction efficiency and the respective UV-Vis analysis. Table 6 shows the values of ssDNA concentration, 260/280 ratio, total yield, and extraction efficiency. The results indicate the impact of Si NPs on the polymer improves the properties of the polymer, although the conditions are same as before adding the binding buffer. The binding buffer (salt + alcohol, salt or alcohol) like GuHCl possess chaotropic properties. The binding buffers play an important role in destabilizing hydrogen bonds in non-polar media. As the hydrogen bonding becomes stronger, the GuHCl/EtOH buffer increases the chemical polarity of the solvent. The increase in chemical polarity destabilizes hydrogen bonding by decreasing the water activity, resulting in insufficient water molecules to effectively solvate the ions. It also disrupts the association of nucleic acids with water. Ethanol was added to influence and enhance the binding of nucleic acids to the Si and correct its concentration to wash off the salts from the membrane [38,39]. 

The comparison of extraction efficiency among the three buffers shown in Figure 8d indicates the efficiency with order GuHCl > NaCl > PBS, which is in accordance with the choatropic strength order. A comparison of the maximum extraction efficiency of the pure polymer with poly(4,4′-cyclohexylidene bisphenol oxalate)/Si NCs is provided in Table 7. A previous study observed lower adsorption for DNA at 1.3 (mg/g) = 1300 ng/μL [40]. In this study, the authors used three NCs of polypyrrole/Si (unfunctionalized PPy/Si, aminated PPy/Si and carboxylated PPy/Si particles) and the best interpretation for enhancing DNA adsorption. It is a function group, where DNA adsorption most likely occurs via both hydrophobic interaction and electrostatic. The hydrogen bonding interactions between the DNA and the surface functional groups probably enhances the strength of carbonyl in poly(4,4′-yclohexylidene bisphenol oxalate)/Si NCs. The modification of poly(4,4′-cyclohexylidene bisphenol oxalate)/Si NCs has a sufficient affinity to capture the ssDNA. The differences of ssDNA purification and total yield amounts depend on the properties of adsorbent such as functional groups, structure, pore size of particles, surface area, and ligand density. It was observed that in the band of UV-Vis, the values of absorbance ratios were investigated to indicate high purity of DNA with higher extraction efficiency (Figure 8a–c).

## 4. Conclusions

The present study used Si NPs for modifying the poly(4,4′-cyclohexylidene bisphenol oxalate) polymer by means of two different methods: Direct solution mixing and in situ polymerization. The physico-chemical properties of the formed NCs were thoroughly studied by means of SEM, DSC, DTC-TGA, FTIR, XRD, and surface area analysis. The DNA extraction studies for the poly(4,4′-cyclohexylidene bisphenol oxalate/Si NC indicated a high efficiency for 2M GuHCl/ethanol of binding buffer. The absorbance ratios of A260/A280 at 0.2 g of polymer/Si NC weight were 2.1, 2.3, and 2.2 for 2 M GuHCl/EtOH, PBS, NaCl, respectively. This indicated highly purified DNA following the extraction process. Following analysis and comparisons with previous literature, the present study has confirmed that the repaired composite was superior to many different materials applied for the DNA extraction study. 

## Figures and Tables

**Table 1:** Comparison of different adsorbent materials along with their advantages and disadvantages.

Material	Advantages	Disadvantages	Typical Yield of DNA	Reference
Glass particles	Sensitive, simple, and reproducible	Requirement of large equipment and high cost.	--	[21]
Silica-based matrices and particles	Easy to perform, and reproducible, high-purity DNA,	Use one-time, unable to recover small DNA fragments; dependence on the DNA fragment sizes.	4–12 μg (blood)-/25–50 (buffy coat)/15–20 (cells) and 9.0 mg/g.	[22,23]
Magnetic nanoparticles-based DNA purification	Equipment-free, no centrifugation, best choice for automation,	Interference in PCR amplification	18–35 μg DNA	[24]
Diatomaceous earth	Shorter protocol; Reduced pipetting error	High cost	Up to 40 μg	[25]
Anion-exchange material	Reusable resins	Presence of high-salt concentrations	350 μg	[26]
Cellulose matrix	Easy to use and storage	Extraction protocols being prone to error and complex.	1–5 μg (plant) 1–3 μg (dried blood spots)	[27,28]

**Table 2:** Amount of Si NPs used in the experimental and final yield of poly(4,4′-cyclohexylidene bisphenol oxalate)/Si nanocomposites (NCs).

Run	Si NPs (wt.%)	Polymer NCs Yield (%)
1	3.7	28.0
2	7.0	68.0
3	13.0	88.3

**Table 3:** Comparison of the FTIR spectral band positions of poly(4,4′-cyclohexylidene bisphenol oxalate)/Si NCs with different ratios of Si.

FTIR Band Type in Poly(4,4′-Cyclohexylidene Bisphenol Oxalate)/Si NC	Poly(4,4′-Cyclohexylidene Bisphenol Oxalate)/Si(3.7%)	Poly(4,4′-Cyclohexylidene Bisphenol Oxalate)/Si(13%)	Poly(4,4′-Cyclohexylidene Bisphenol Oxalate)/Si(7%)	Poly(4,4′-Cyclohexylidene Bisphenol Oxalate)/Si Solution
C=O stretching	1603.31 cm^−1^	1603.70 cm^−1^	1603.76 cm^−1^	1608.10 cm^−1^
C–H stretching	3257.96 cm^−1^	3173.58 cm^−1^	3182.34 cm^−1^	3157.59 cm^−1^
Si–O stretching	1074.72 cm^−1^	1177.20 cm^−1^	1176.61 cm^−1^	1081.93 cm^−1^

**Table 4:** Summary of wide-angle XRD peaks, and corresponding d-spacing for the pure Si NPs and poly(4,4′-cyclohexylidene bisphenol oxalate)/Si NCs with different ratios of Si.

Sample	2*θ*	d-Spacing (Å)	FWHM	Crystalized Size (nm)
Poly(4,4′-cyclohexylidene bisphenol oxalate)/Si solution mixture	22	4.033	0.059	137.277
Poly(4,4′-cyclohexylidene bisphenol oxalate)/Si(3.7%)	21	5.802	0.236	33.936
Poly(4,4′-cyclohexylidene bisphenol oxalate)/Si(7%)	21	5.843	0.295	27.152
Poly(4,4′-cyclohexylidene bisphenol oxalate)/Si(13%)	21	5.850	0.2755	29.092
Pure Si NPs	21	--	--	--

**Table 5:** Comparison of the BET and BJH (Barrett–Joyner–Halenda method) results.

Polymer/Si Ratio	BET Surface Area (m^2^/g)	BJH Adsorption Surface Area (m^2^/g)	BJH Desorption Surface Area (m^2^/g)	BJH Adsorption Pore Volume (cc/g)—Pore Radius Dv (r) (Å)	BJH Desorption Pore Volume(cc/g)—Pore Radius Dv (r) (Å)
Poly(4,4′-cyclohexylidene bisphenol oxalate)/Si(3.7%)	1.788	2.740	3.718	0.059–28.393	0.060–155.260
Poly(4,4′-cyclohexylidene bisphenol oxalate)/Si(7%)	3.361	5.898	6.681	0.073–159.228	0.073–149.427
Poly(4,4′-cyclohexylidene bisphenol oxalate)/Si(13%)	12.237	22.317	27.576	0.282–15.418	0.288–156.464
Poly(4,4′-cyclohexylidene bisphenol oxalate)/Si solution maxing 40 wt.%	76.947	120.354	151.621	1.810–148.713	1.828–153.888

**Table 6:** Extraction efficiencies and total yields of DNA for the poly(4,4′-cyclohexylidene bisphenol oxalate)/Si (13%) NC when tested under different weights and binding buffers.

Binding Buffer	Polymer Weight	Concentration	260/280	Total Yield (ng)	Extraction Efficiency (ng/μL)
2 M GuHCl/EtOH	0.2 g	1348	2.1	1,348,000	3370
PBS	0.2 g	365	2.3	165,000	913
2 M NaCl	0.2 g	713	2.2	713,000	1783
2 M GuHCl/EtOH	0.1 g	965	1.6	482,500	2413
PBS	0.1 g	342	2.6	171,000	855
2 M NaCl	0.1 g	408	2.3	204,000	1020
2 M GuHCl/EtOH	0.02 g	740	1.7	370,000	1850
PBS	0.02 g	152	1.5	76,000	380
2 M NaCl	0.02 g	332	1.8	166,000	830

**Table 7:** Comparison of the extraction efficiencies of pure polymer, poly(4,4′-cyclohexylidene bisphenol oxalate), with that of poly(4,4′-cyclohexylidene bisphenol oxalate)/Si(13%) NC.

Binding Buffer	Sample Weight	Pure Polymer Extraction Efficiency (ng/μL)	poly(4,4′-Cyclohexylidene Bisphenol Oxalate)/Si(13%) NC Extraction Efficiency (ng/μL)
2M GuHCl/EtOH	0.2 g	2248	3370
PBS	0.2 g	875	913
2 M NaCl	0.2 g	1693	1783
2M GuHCl/EtOH	0.1 g	2080	2413
PBS	0.1 g	805	855
2 M NaCl	0.1 g	993	1020
2M GuHCl/EtOH	0.02 g	1583	1850
PBS	0.02 g	129	380
2 M NaCl	0.02 g	315	830
